# Bioprinted Recombinant Human-Collagen-Based In Vitro Skin Models for Assessing Effects of Nano-ZnO on Dermis

**DOI:** 10.3390/gels11120977

**Published:** 2025-12-04

**Authors:** Ting Yu, Yang Xu, Xinyue Zhang, Chenkai Zhu, Jianfeng Zhao, Yang Yang, Min Jiang

**Affiliations:** 1 State Key Laboratory of Materials-Oriented Chemical Engineering, College of Biotechnology and Pharmaceutical Engineering, Nanjing Tech University, Nanjing 211800, China; 2 Ningbo Institute of Technology, Beihang University, 399 Kangda Road, Ningbo 315832, China; 3 Zhejiang Zhuji Jland Biotechnology Co., Ltd., No.69 Youyi North Road, Zhuji 311800, China

**Keywords:** in vitro skin model, 3D bioprinting, recombinant human collagen, nano-zinc oxide, vascularization potential

## Abstract

Collagen types I and III are primary structural proteins that maintain human skin integrity, and their ratio is disrupted during aging. In this study, we developed a biomimetic 3D skin model with vascularization potential to evaluate the effects of nano-zinc oxide (nano-ZnO) in a physiologically relevant context. The model used methacrylated recombinant human collagen (RHC-MA) bioinks with tunable collagen I/III ratios that mimic the skin of children and adults. The bioinks exhibited excellent printability and mechanical properties that enabled the 3D bioprinting of full-thickness skin products, including dermal layers with human skin fibroblasts (HSFs) and human umbilical vein endothelial cells (HUVECs), as well as epidermal layers with keratinocytes (KCs). The model recapitulated native skin architecture, and key markers such as keratin 10 (K10), keratin 14 (K14), and cluster of differentiation 31 (CD31) were determined. Quantitative real-time polymerase chain reaction (qRT-PCR) analysis showed that nano-ZnO significantly modulated genes associated with apoptosis, inflammation, and oxidative stress in skin cells.

## 1. Introduction

Skin is the largest organ of the human body, and the maintenance of its structure and function depends on the collagen network of the extracellular matrix (ECM) [1,2]. Type I collagen (accounting for 80–90% of total) provides mechanical strength and support, while type III collagen (10–20%) contributes to skin’s elasticity and flexibility [3]. Interestingly, the high collagen III levels that grant infant skin its softness and regenerative capacity give way to a predominance of collagen I during aging, leading to wrinkles and sagging [4]. In addition, things like daily habits and environmental factors predispose skin to protein loss and even raise the risk of cancer [5]. Consequently, specific functional ingredients are often added to cosmetics and skincare products to protect the skin.

Nano-zinc oxide (nano-ZnO) is widely used in cosmetics [6] and skin wound treatments [7], mainly because of its remarkable ability to fight bacteria and reduce inflammation [8]. In cosmetics, it serves as a key ultraviolet (UV) filter in sunscreen, wherein it works by reflecting and scattering ultraviolet rays to prevent sun damage [9]. Xiao et al. summarized nano-ZnO’s multifaceted functions, such as its antimicrobial, anti-inflammatory, and pro-angiogenic properties, in relation to pathologies relating to mechanical injuries, diabetic ulcers, and burns in skin tissue regeneration [7]. Additionally, Zhu et al. found that nano-ZnO can selectively and effectively eradicate Cutibacterium acnes, the acne-causing bacterium, while preserving normal skin microbiota [10]. Although it is classified as safe by the US Food and Drug Administration (FDA) and used in medical applications such as drug delivery, tissue regeneration, and bioimaging, nano-ZnO is still regarded as a toxic metal oxide nanoparticle [11]. Zhang et al. studied the toxic effect of nano-ZnO on human umbilical vein endothelial cells (HUVECs) and found that nano-ZnO significantly reduced cell viability and induced cell death [12]. The authors also reported that nano-ZnO disrupts iron metabolism, increases mitochondrial fission, and disturbs redox homeostasis, ultimately triggering ferroptosis. Similarly, Yin et al. observed that nano-ZnO induces concentration-dependent cytotoxicity, ROS accumulation, and mitochondrial dysfunction in human keratinocytes [13]. Sharma et al. further demonstrated that nano-ZnO sharply increases intracellular reactive oxygen species (ROS) levels in human hepatocytes, thereby inducing oxidative stress, DNA damage, and cell death via the mitochondria-mediated apoptotic pathway [14]. To date, our understanding of the effects of nano-ZnO on skin mainly derives from direct cell attachment or solution exposure. These approaches constitute basic cytotoxicity assessments in two-dimensional (2D) cell cultures and, to date, have not been comprehensively evaluated in biomimetic, three-dimensional (3D) skin structures, especially the dermal layers with vascularization capacity. Therefore, establishing a 3D in vitro model that accurately mimics real skin’s components, structure, and function is critical.

Tissue-engineered skin models are biological products with skin-like structures and functions. They are developed in vitro through co-culturing of skin cells and biomaterials under three-dimensional conditions [15]. These models apply to fields like drug testing and disease modeling [16]. Moreover, they align with the European “3R principles” (replacement, reduction, and refinement) by replacing animal experiments [17]. Cavallo et al. developed a bilayer skin model using marine collagen and alginate bioink with human fibroblasts and keratinocytes via extrusion-based 3D bioprinting [18]. Ma et al. fabricated artificial skin using gelatin-based hydrogel bioink with epidermal cells via 3D bioprinting [19]. Fauzi et al. developed a skin model using ovine type I collagen mixed with keratinocytes and fibroblasts [20]. Zhou et al. used gelatin and hyaluronic acid as bioinks and employed 3D bioprinting technology based on digital light processing (DLP) to construct a double-layer skin model including the epidermis and dermis [21]. However, the above biomaterials are mainly animal-derived products, and dermal layers with endothelial cells are commonly neglected.

**Figure 1:**
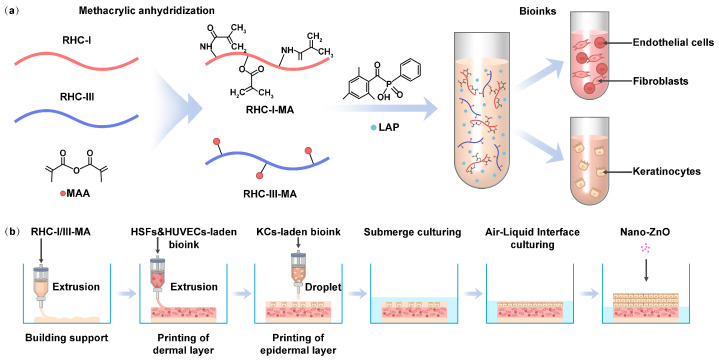
Schematic diagram of the bioprinted in vitro 3D skin model made from RHC-based bioinks. (**a**) Preparation of the cell-laden bioinks. (**b**) The 3D bioprinting of the in vitro skin model using extrusion and droplet modes.

Thus, replicating the natural content and structure of human skin is crucial for preparing in vitro skin models. In this study, we use recombinant human collagen (RHC) as the primary biomaterial, as its amino acid sequence closely matches that of human collagen [22]. RHC is much safer than animal-derived collagen, which poses risks of immunogenicity, viral infection, and unclear structure–function relationships [23,24]. Additionally, previous studies confirmed that RHC promotes skin fibroblasts’ adhesion, proliferation, and migration, highlighting its potential for tissue repair and regeneration [25]. Furthermore, adjusting the recombinant collagen I/III ratio to match age-related skin compositions allows custom skin models to mimic specific age groups. Subsequently, 3D bioprinting can be used to precisely print full-thickness skin products with a dermal layer of fibroblasts and endothelial cells and an epidermal layer of keratinocytes (KCs) [26]. A schematic diagram of preparing the in vitro 3D skin model is shown in Figure 1. In this study, we simulated and validated RHC grafting with methacrylic anhydride (MAA) using Fourier transform infrared spectroscopy (FTIR), proton nuclear magnetic resonance (^1^H-NMR), and the 2,4,6-trinitrobenzenesulfonic acid (TNBS) method. The mechanical properties of the RHC-based bioinks, along with their photorheological properties, were investigated through compression and fatigue testing. Finally, the dermis and epidermis of the in vitro model were printed using the intrusion and droplet modes of the bioprinter, respectively. The maturation and differentiation of the epidermis were achieved through air–liquid culturing. The effects of nano-ZnO on the dermis were thus investigated through this 3D bioprinted in vitro skin model.

## 2. Results and Discussion

### 2.1. Methacrylation of Recombinant Human Collagen

Although RHC is a non-photosensitive biomaterial, its molecular chain contains active functional groups such as amino, carboxyl, and hydroxyl groups, providing potential sites for chemical grafting modification [27]. To endow it with photo-responsive capacity, we modified RHC with MAA, and the methacryloyl group was successfully added. This modification introduces vinyl groups into RHC molecules. These groups serve as key functional groups for photo-initiated crosslinking reactions [28].

#### 2.1.1. FTIR

**Figure 2:**
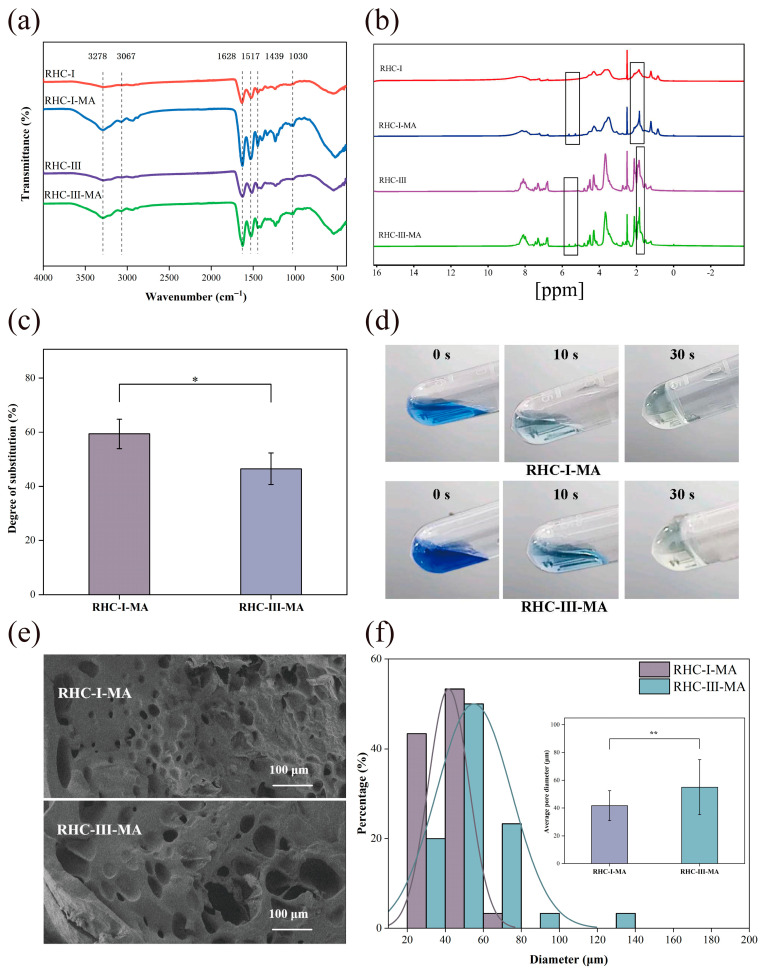
Characterization of RHC-I and RHC-III methacrylation. (**a**) FTIR spectra of RHC before and after MAA grafting. (**b**) ^1^H-NMR spectra of RHC before and after MAA grafting. (**c**) Grafting ratios of RHC-I-MA and RHC-III-MA. Significance is indicated with * *p* < 0.05, with n = 3. (**d**) UV-cured bioinks. (**e**) SEM images of the hydrogels. Scale bar = 100 µm. (**f**) The pore size distribution and average pore size of the hydrogels. Significance is indicated with ** *p* < 0.01, with n = 3.

As shown in Figure 2a, the broad band at 3278 cm^−1^ can be attributed to the stretching vibrations of the amino (-NH) and hydroxyl (-OH) groups in the amide regions of RHC [29]. Upon the addition of MAA, this absorption peak intensified, a response attributable to ester bond formation via the anhydride reaction between MAA and amine groups in RHC repeating units. The C-H stretching vibration peak of RHC at 3067 cm^−1^ shifted following MAA introduction. The bands at 1628 cm^−1^ and 1517 cm^−1^ arose from the C=O stretching vibration of amide I and the N-H deformation of amide II, respectively, in both RHC spectra [30]. After the reaction with MAA, both peaks markedly increased. The peak at 1439 cm^−1^ corresponds to C-N stretching vibrations of the amide bond, while the peak at 1030 cm^−1^ is associated with in-plane C=C-H bending vibrations. The in-plane C=C-H bending vibration at 1030 cm^−1^ was more pronounced in RHC-MA than in RHC, serving as a characteristic peak indicative of methacrylic substitution after the grafting of MAA onto RHC. These findings confirm that MAA was successfully grafted onto both RHC-I and RHC-III.

#### 2.1.2. ^1^H-NMR

As shown in Figure 2b, new peaks appeared at chemical shifts of 5.30 and 5.63 ppm in both RHC-MA variants. These peaks originated from the acrylic protons (2H) of the grafted methacryloyl groups. The peak at 1.84 ppm arose from the methyl protons (3H) of the grafted methacryloyl groups [31,32]. These findings indicate that the methacryloyl groups were successfully grafted onto the RHC molecules.

#### 2.1.3. Substitution Degree

The MAA substitution degrees for RHC-I-MA and RHC-III-MA were determined using a TNBS assay. As shown in Figure 2c, the substitution degrees were 59.37% for RHC-I-MA and 46.51% for RHC-III-MA. The TNBS results demonstrate successful methacrylation of both RHC-I and RHC-III with MAA. The photo-crosslinking results (Figure 2d) confirm that RHC-I-MA and RHC-III-MA gelled within 30 s of UV exposure, indicating the photosensitivity of the fabricated RHC-MA. The modification of RHC with methacryloyl groups, confirmed via FTIR, TNBS, and ^1^H-NMR, endowed the RHC-MA with rapid photo-curing capacity (within 30 s), facilitating its transition into a printable gel.

### 2.2. Internal Morphological Structure

The internal structure of a hydrogel is closely related to its physical and mechanical properties [33]. In addition, a hydrogel’s microstructure, e.g., pore size, shape, connectivity, and network distribution, also has a crucial impact on cell behavior and tissue formation [34]. The internal morphologies of the UV-cured bioinks were characterized via SEM, and the results are shown in Figure 2e,f. All the RHC-MA samples exhibited porous characteristics. Figure 2f shows that the pore sizes of RHC-I-MA and RHC-III-MA range from 20 to 130 μm, with average pore sizes of 41.78 μm and 55.11 μm, respectively. Evidently, the pores of the type I collagen bioink are smaller than those of the type III collagen bioink, wherein the reduction in pore size is usually negatively correlated with mechanical properties [35,36].

### 2.3. Photorheological Characterization

**Figure 3:**
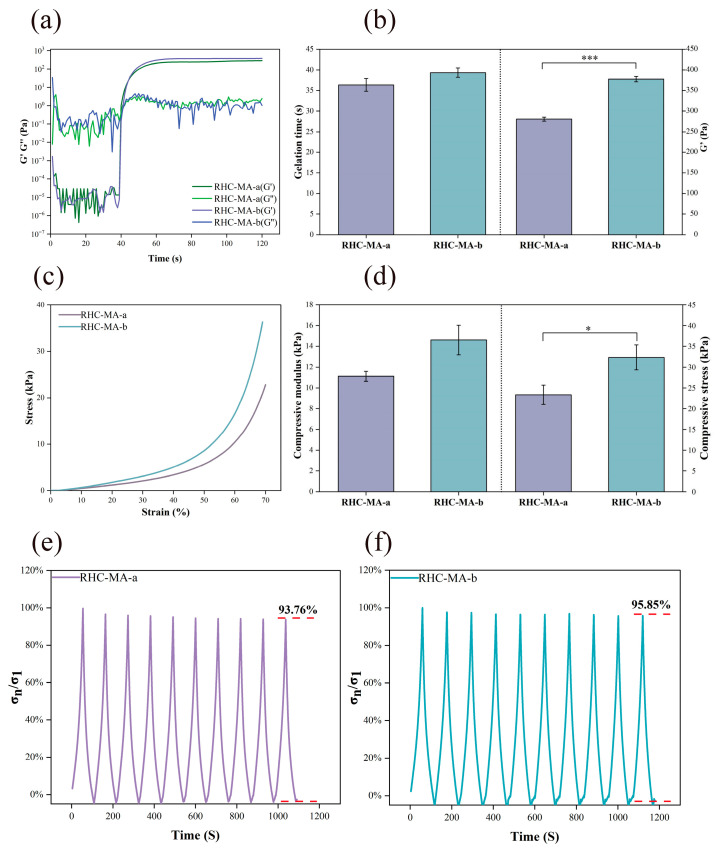
Analysis of the photorheological and mechanical properties of the RHC-based composite hydrogels. (**a**) The storage and loss moduli of the composite hydrogels determined during UV exposure. (**b**) Gelation time and stabilized G’ from the photorheological test. Significance is indicated with *** *p* < 0.001, with n = 3. (**c**) Representative stress–strain curves of the composite hydrogels. (**d**) Compressive modulus and compressive stress of the composite hydrogels. Significance is indicated with * *p* < 0.05, with n = 3. (**e**) The compressive stress ratio of σ_n_/σ_1_ of RHC-MA-a as a function of time over 10 cycles. (**f**) The compressive stress ratio of σ_n_/σ_1_ of RHC-MA-b as a function of time over 10 cycles.

The photorheological properties of bioinks are crucial for ensuring their printability, shape fidelity, and structural stability while maintaining cell viability and biocompatibility [37]. The photorheological properties of RHC-MA-a and RHC-MA-b were investigated by recording their storage modulus (G’) and loss modulus (G”). Time scan curves of the two samples are shown in Figure 3a. Both bioinks exhibited typical gel transformation characteristics, and G’ tended to stabilize after approximately 60 s, indicating a change in the morphology of the hydrogel. Figure 3b shows that the gelation times of RHC-MA-a and RHC-MA-b are 36.3 s and 39.3 s, with no significant differences. This finding indicates that under 405 nm ultraviolet irradiation, all the RHC-MA samples underwent rapid solidification within 40 s. The final G’ values of RHC-MA-a and RHC-MA-b were 280.6 Pa and 377.3 Pa, respectively, indicating that their mechanical strength significantly improved after gelation. This feature also proves that the selected RHC I/III ratios of these two composite hydrogels are close to the ratio for natural skin, as they have ideal elastic properties, making them suitable for the 3D bioprinting of skin models [38].

### 2.4. Mechanical Property Analysis

Bioprinted materials should have adequate mechanical properties, as insufficient mechanical strength can lead to collapse or interlayer slippage after printing [39]. Representative stress–strain curves of RHC-MA-a and RHC-MA-b are shown in Figure 3c. Both samples exhibited typical viscoelastic behaviors that are similar to those of human tissues, and the results reflect the material’s ability to resist tensile or compressive deformation [40]. The compressive moduli of RHC-MA-a and RHC-MA-b were found to be 11.1 and 14.6 kPa, while the compressive stresses at 70% strain were 23.3 and 32.3 kPa, respectively (Figure 3d). These results demonstrate that RHC-MA-b-formed hydrogel is more capable of resisting compression deformation. They also reflect the greater stiffness of the mimicked adult skin formula [41].

In addition, the long-term stability and durability of the RHC-MA-a and RHC-MA-b hydrogels were further evaluated through compressive fatigue testing under simulated physiological conditions. As shown in Figure 3e,f, the peak σ_n_/σ_1_ ratios remained stable with no significant degradation after 10 compression cycles, indicating robust fatigue resistance in both formulas. In summary, the composite hydrogels exhibit satisfactory mechanical properties suitable for subsequent skin model construction.

### 2.5. Printability and Cell Survivability

**Figure 4:**
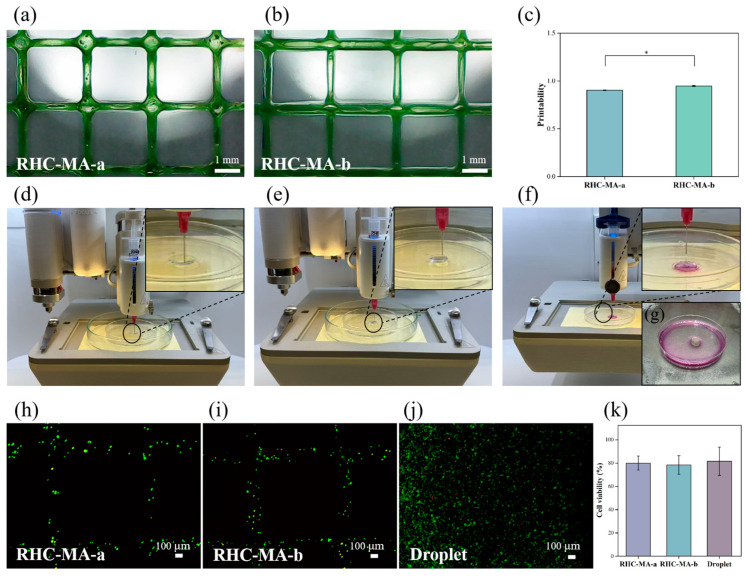
The printability of the bioinks and the construction of the 3D skin model. (**a**) Regular grids printed using RHC-MA-a. Scale bar = 1 mm. (**b**) Regular grids printed using RHC-MA-b. Scale bar = 1 mm. (**c**) Semi-quantitative evaluation of printability for the two bioinks. Significance is indicated with * *p* < 0.05, with n = 3. (**d**) Printing the support base in extrusion mode. (**e**) Printing the dermis containing HSFs and HUVECs in extrusion mode. (**f**) Printing the epidermal layer containing KCs in droplet mode. (**g**) The final 3D skin model, printed. (**h**,**i**) LIVE/DEAD™ staining of the bioprinted cell-laden products. Scale bar = 100 µm. (**j**) LIVE/DEAD™ staining of the cells in the droplet-formed product. Scale bar = 100 µm. (**k**) Cell survivability of the printed products.

Printability is a key prerequisite for a bioink in 3D bioprinting. It directly affects the structural integrity of the product and is closely related to the adhesion and proliferation of cells [42,43]. The printed grids and determined printability of bioinks are shown in Figure 4a–c. Although the printing capacity of RHC-MA-a slightly decreased with the increase in the proportion of collagen I, the printability (Pr) values of both bioinks remained between 0.9 and 1.1. This finding suggests that the RHC-based bioinks can endow printed products with ideal structural fidelity and mechanical stability [44].

Figure 4d–g show the complete process of manufacturing the in vitro skin model through 3D bioprinting, confirming the excellent printing performance of the bioink. Initially, the RHC-MA support base (Figure 4d) and the dermis containing human skin fibroblasts (HSFs) and HUVECs (Figure 4e) were prepared using the extrusion printing method. Subsequently, the KCs were deposited onto the dermis using the droplet printing method to form the epidermis (Figure 4f). This bioink ensured different cell types had a precise spatial distribution, generating a well-structured and laminated multilayer skin model (Figure 4g). The obtained 3D skin model maintained a complete structure and stable shape, without deformation or collapse. The cell viabilities of the printed hydrogel lattice products and the droplet-formed cell-laden products were examined using LIVE/DEAD™ staining (Figure 4h–j). The results clearly show that the majority of the cells were alive, with the cell survivability (Figure 4k) of the selected bioprinted products ranging from 78% to 82%.

### 2.6. Histological Examination of In Vitro 3D Skin Models

**Figure 5:**
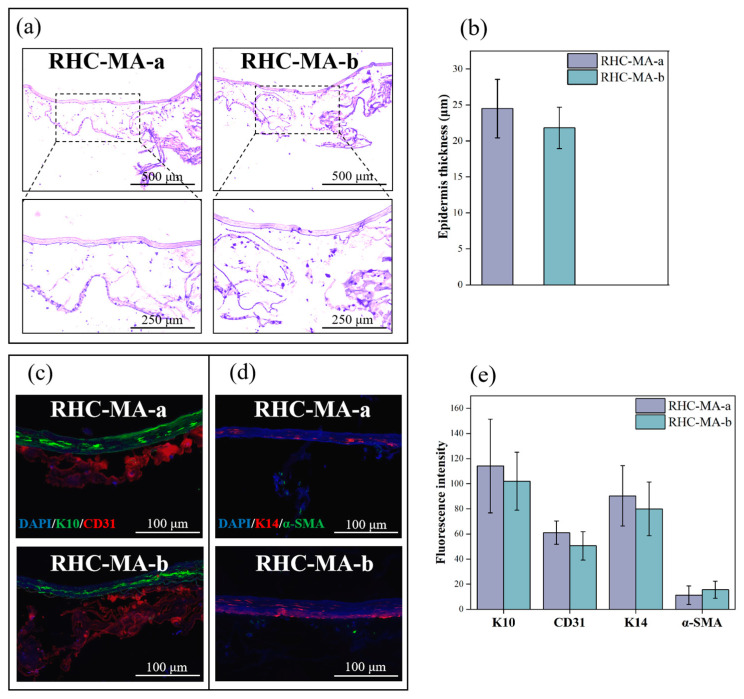
Histological characterization of the in vitro skin models. (**a**) H&E staining of the skin models. Scale bars: 500 µm (upper panels) and 250 µm (lower panels). (**b**) Thickness of the epidermis of the skin products (n = 3). (**c**) Immunohistochemical images of epidermal layers, showing merged DAPI (blue), K10 (green), and CD31 (red). Scale bar = 100 µm. (**d**) DAPI (blue), K14 (red), and α-SMA (green). Scale bar = 100 µm. (**e**) Fluorescence intensity pertaining to the expression of K10, CD31, K14, and α-SMA from epidermal layers (n = 3).

Recombinant collagen shows great potential in tissue engineering due to its excellent biocompatibility, customizable mechanical properties, and ability to enhance cell proliferation and differentiation [45]. Studies indicate that applying RHC in the construction of skin models can create a favorable environment for cell growth and tissue development [46]. Hematoxylin and eosin (H&E) staining was used to illustrate the properties of the bioprinted full-thickness in vitro skin models. As shown in Figure 5a, H&E staining indicated that the 3D bioprinted skin structure contained proliferating basal layers and a differentiated suprabasal epidermis. We further quantified the epidermal thickness of RHC-MA-a and RHC-MA-b and found no significant differences, with the average thickness ranging from 21.83 to 24.50 µm (Figure 5b). Meanwhile, the HSFs and HUVECs were homogenously distributed in the dermis. The entire in vitro tissue structure is clearly visible and very similar to natural human skin.

In addition, the skin products were immunohistochemically stained to determine the functionality of the in vitro skin model. As shown in Figure 5c, intense, continuous green fluorescence (K10) was observed in both RHC-MA groups, indicating obvious terminal differentiation of epidermal cells. Importantly, red fluorescence (CD31) was clearly observed in both samples, demonstrating the expression of vascularization-associated protein from endothelial cells and confirming the printed dermal layers had the capacity to vascularize [47]. DAPI staining showed a dense blue color, indicating distribution of HSFs and HUVECs in dermal layers. Furthermore, in Figure 5d, the red fluorescence (K14) was significantly weakened, indicating a decrease in the proliferative activity of basal cells and suggesting that the epidermis had begun transitioning from the proliferative stage to the mature and stable stage. Weak green fluorescence (α-SMA) indicated low myofibroblast activity and a low risk of tissue fibrosis. We quantified the fluorescence intensities of the markers K10, CD31, K14, and α-SMA and found no significant differences between the two model groups (Figure 5e). These findings demonstrate that these RHC-based materials could support cell proliferation and differentiation, as key proteins of the dermal and epidermal layers were expressed and the materials exhibited low fibrotic potential. Together, these results confirm that both the child and adult skin models effectively recapitulate the key biological characteristics and structures of native skin.

### 2.7. Gene Expression Analysis

**Figure 6:**
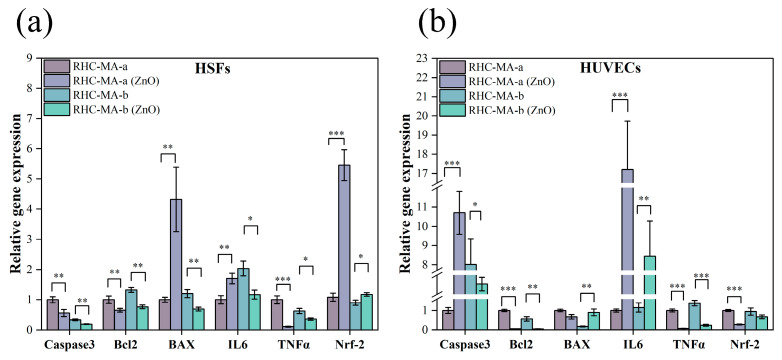
Effects of nano-ZnO on gene expression in dermal cells. (**a**) Gene expression of Caspase3, Bcl2, BAX, IL-6, TNF-α, and Nrf2 in HSFs in the dermal layer of the in vitro skin models. Significance is indicated with * *p* < 0.05, ** *p* < 0.01, and *** *p* < 0.001, with n = 3. (**b**) Gene expression of Caspase3, Bcl2, BAX, IL-6, TNF-α, and Nrf2 in HUVECs in the dermal layer of the in vitro skin models. Significance is indicated with * *p* < 0.05, ** *p* < 0.01, and *** *p* < 0.001, with n = 3.

To examine the effects of nano-ZnO on the dermal layer of the skin at the genetic level, we used qRT-PCR to analyze the expression of key genes in the prepared skin models. As shown in Figure 6a, after treatment with nano-ZnO, the expression of the apoptotic gene Caspase3, the anti-apoptotic gene Bcl2, and the pro-inflammatory cytokine TNF-α decreased in HSFs of both skin models. The expression of Nrf2 was upregulated in both samples; Nrf2 is known to be a core transcription factor that regulates the antioxidant and anti-stress responses of cells [48]. In the RHC-MA-a sample, the expression of the pro-apoptotic gene BAX and the inflammatory factor IL-6 also increased after treatment with nano-ZnO. Previous studies confirmed that key genes such as Caspase-3, Bcl2, and BAX constitute core components of the mitochondrial apoptotic pathway, finely tuning cellular apoptotic capacity [49,50]. As shown in Figure 6b, for the HUVECs in the dermis, the addition of nano-ZnO to the two samples could significantly reduce the expression of Bcl2, TNF-α, and Nrf2. Previous studies have confirmed that IL-6 and TNF-α can trigger inflammatory responses and recruit immune cells [51,52]. Accordingly, our results demonstrate that nano-ZnO interfered with the gene expression of dermal cells in our skin models. Interestingly, through comparison with the adult skin model (RHC-MA-b), we noticed the child skin model (RHC-MA-a) was more likely to induce the expression of BAX, IL-6, and Nrf2 in HSFs as well as Caspase3 and IL-6 in HUVECs. Conversely, the adult skin model displayed enhanced anti-apoptotic and anti-inflammatory capacities, as reflected by Bcl2 and TNF-α expression patterns. These differences may stem from variations in the physiological structure and cellular metabolic characteristics between child and adult skin [53].

## 3. Conclusions

We effectively developed composite bioinks of RHC-I and RHC-III in varying ratios and combined them with skin cells (HSFs, HUVECs, and KCs) to fabricate in vitro skin models with vascularization potential via 3D bioprinting. These models were used to assess the impact of nano-ZnO on models representing child (RHC-MA-a) and adult (RHC-MA-b) skin. Photorheological and mechanical assessments validated that RHC-MA-a and RHC-MA-b bioinks exhibit favorable printability and rapid photo-curing properties, ensuring a smooth printing process, precise structural integrity, and mechanical stability of the skin models. Histological and immunohistochemical analyses revealed that both the child and adult skin models could accurately reproduce the multi-layered epidermal structure of natural skin, express key proteins (K10, K14, and CD31), and achieve functional differentiation from cell proliferation to tissue maturation. The models’ structures and biological characteristics were highly similar to those of in vivo skin. Finally, gene expression analysis further indicated that nano-ZnO could regulate the apoptotic, inflammatory, and oxidative stress responses of skin cells in child and adult skin models.

## 4. Materials and Methods

### 4.1. Materials and Reagents

Type I and III recombinant human collagen (RHC-I, RHC-III) were obtained from JLand Biotech Co., Ltd. (Zhuji, China). MAA was purchased from Aladdin (Shanghai, China). Lithium phenyl-2,4,6-trimethylbenzoylphosphinate (LAP) and nano-ZnO, with a particle size of 50 ± 10 nm, were purchased from Macklin (Shanghai, China). Dimethyl sulfoxide (DMSO) was purchased from Cambridge Isotope Laboratories, Inc. (Cambridge, UK). 2,4,6-trinitrobenzenesulfonic acid (TNBS) was purchased from Acmec Biochemical Co., Ltd. (Shanghai, China). Phosphate-buffered saline (PBS) was purchased from Wisent (Saint Sauveur, QC, Canada). HSFs, HUVECs, and KCs were purchased from Zhongqiao Xinzhou Biotechnology Co., Ltd. (Shanghai, China).

### 4.2. Preparation of the Bioink Materials and Reagents

First, 1 g of RHC-I powder and 1 g of RHC-III powder were separately weighed and dissolved in PBS to prepare 10% (*w*/*v*) RHC-I and RHC-III solutions, respectively. Subsequently, a 2% (*v*/*v*) MAA solution was added dropwise to the RHC-I and RHC-III solutions to obtain RHC-I-MA and RHC-III-MA solutions, respectively. The mixtures were stirred at room temperature (24 °C) for 5 h to ensure complete reaction. The solutions were then centrifuged at 7000 rpm for 8 min. The supernatant was collected, diluted twofold with pure water, and transferred into a dialysis bag (MV 14000, Biosharp, Tallinn, Estonia). Dialysis was performed at room temperature for 3–5 days to remove unreacted MAA. After dialysis, the solutions were sterilized via filtration through a 0.22 μm membrane and lyophilized to obtain RHC-I-MA and RHC-III-MA powder. The RHC-I-MA and RHC-III-MA powders were mixed in different mass ratios (according to the I/III collagen contained in human skin) and dissolved in a PBS solution containing the photo-initiator lithium phenyl-2,4,6-trimethylbenzoylphosphinate (LAP). A list of the samples of the composite RHC-MA, which was used to create printable inks, is shown in Table 1.

### 4.3. Determination of MAA Grafting Ratio in RHC-MA Solutions

#### 4.3.1. Fourier Transform Infrared Spectroscopy (FTIR)

FTIR was used to analyze the functional groups and structural characteristics of the RHC samples. The dry RHC-I, RHC-III, RHC-I-MA, and RHC-III-MA powders were tested using a spectrometer (Nicolet iS5, ATR iD7, Thermo Scientific, Waltham, MA, USA). All spectra were recorded in absorption mode with a scan resolution of 2 cm^−1^ over a wavenumber range of 4000–400 cm^−1^.

#### 4.3.2. Proton Nuclear Magnetic Resonance Spectroscopy (^1^H-NMR)

^1^H-NMR was used to acquire structural information on the chemical environments, quantity, and connectivity of hydrogen atoms in the samples. RHC-I, RHC-III, RHC-I-MA, and RHC-III-MA were separately dissolved in deuterated DMSO to prepare 100 µg/mL solutions. The ^1^H chemical shifts of each sample were observed using an NMR spectrometer (Avance III 500 MHz, Bruker, Fällanden, Switzerland) to detect new peaks corresponding to MAA, thus confirming the grafting reaction.

#### 4.3.3. TNBS Assay

The substitution degree for the RHC-MA molecules was determined using the TNBS assay. Firstly, glycine standard solutions (0, 0.8, 8, 16, 32, and 64 µg/mL, respectively) and a 0.2% (*w*/*v*) TNBS aqueous solution were prepared, and the standard curves were plotted. The RHC and RHC-MA samples were diluted to 1.6 mg/mL in 0.1 M sodium bicarbonate buffer. Then, 500 µL of each sample (RHC and RHC-MA) was mixed with 500 µL of 0.2% TNBS solution and incubated at 37 °C for 3 h. After the reaction, 250 µL of 0.1 M hydrochloric acid and 500 µL of 10% (*w*/*v*) sodium dodecyl sulfate were added to terminate the reaction. Finally, the absorbance of the samples and standards was determined at 335 nm using a microplate reader (Infinite M200 PRO, Tecan, Vienna, Austria). The sample concentrations were calculated by substituting each sample’s absorbance value into the standard curve, and the degree of substitution (DS) was calculated using the formula DS = (C_RHC_ − C_RHC_-_MA_)/C_RHC_, where C represents the concentration from the standard curve.

### 4.4. Morphological Characterization

The hydrogels (RHC-I-MA and RHC-III-MA) were freeze-dried, and their internal structures were observed using scanning electron microscopy (SEM). The samples were cut with a scalpel and then subjected to gold sputter coating using a turbo molecular pump coating instrument (Q150T, Quorum, Laughton, UK). The internal morphologies of the samples were visualized using SEM (Regulus 8230, Hitachi, Tokyo, Japan) under an accelerated voltage of 3 kV. Finally, the pore size distribution and average pore size of RHC-I-MA and RHC-III-MA were analyzed using ImageJ-Fiji software.

### 4.5. Physicochemical Properties of the Hydrogels

#### 4.5.1. Characterization of Photorheological Properties

Photorheological properties provide information on a bioink’s flow behavior and related mechanical properties. The photorheological properties of RHC-MA-a and RHC-MA-b under UV exposure were measured using a rotational rheometer (Anton Paar MCR 302e) equipped with a photo-curing module. The rheometer platform was maintained at 25 °C. A UV lamp was switched on at 30 s, with a total exposure of 60 s. Tests were performed with a shear strain of 1% and an angular frequency of 5 rad/s. The storage modulus (G′) and loss modulus (G″) of the collagen bioink were recorded for up to 120 s throughout the test. The gelation time was defined as the duration from the start of UV exposure to the gel point.

#### 4.5.2. Mechanical Properties

A universal mechanical testing machine (UTM4304X, Suns, Shenzhen, China) was used to perform compression tests on the composite collagen hydrogels (RHC-MA-a, RHC-MA-b). The hydrogels were molded into cylindrical specimens (diameter, 20 mm; height, 10 mm) using polytetrafluoroethylene (PTFE) molds. The samples were compressed at 2 mm/min, and the stress at 70% strain was recorded. The compressive modulus was determined based on the stress–strain curve slope at 10% strain. Afterwards, fatigue testing was conducted by subjecting the samples to 10 repeated compression cycles at a rate of 10 mm/min, with a strain ranging from 0% to 50% and back to 0%. The change in the compression stress ratio (σ_n_/σ_1_) over 10 cycles within 1200 s was recorded for each sample. Here, σ_n_/σ_1_ denotes the ratio of the maximum stress in each cycle (σ_n_) to that of the first cycle (σ_1_).

#### 4.5.3. Investigation of Printability and Cell Survivability

To assess printability, a square grid structure was printed using the mechanical screw extrusion mode of a 3D bioprinter (Bio-X, Cellink, Gothenburg, Sweden) and evaluated. A 25G nozzle was used in printing, with bioink pre-loaded into a 2.5 mL syringe. The printing speed and extrusion volume were set to 5 mm/s and 0.07 µL/mm, respectively. Printed grids were stabilized by exposure to 405 nm UV light for 40 s. Printability (Pr) was calculated as Pr = L^2^/16A, where L is the grid perimeter and A is the enclosed area [54]. Printed grid images were analyzed using ImageJ software. The cell viability of the bio-printed products was visualized using LIVE/DEAD™ (Invitrogen, Carlsbad, CA, USA) staining, and fluorescent images were obtained using an inverted fluorescence microscope (X-81, Olympus, Tokyo, Japan). Cell survivability was analyzed using ImageJ software.

### 4.6. Cell-Laden Bioprinted 3D Skin Products

First, 10% (*w*/*v*) RHC-I-MA and RHC-III-MA solutions were mixed in ratios of 7:3 and 9:1 to prepare bioinks. Before printing, we transferred the bioink into a 2.5 mL syringe equipped with a 25G nozzle. A support base layer of RHC-MA gel (diameter = 10 mm, height = 2 mm) was printed on a culture dish via extrusion (speed: 5 mm/s, volume: 0.07 µL/mm). HSFs and HUVECs were resuspended at 1 × 10^6^ cells/mL in respective RHC-MA bioinks. These cell-laden bioinks were extruded to print the dermal layer onto the support base with the same printing parameter as the support base layer, and these products were immediately crosslinked using 405 nm UV light for 40 s to form the dermal layer. Finally, using droplet-based printing mode (nozzle: 25G, time: 0.5 s, pressure: 20 kPa), bioink containing KCs at 1 × 10^6^ cells/mL was deposited onto the dermal layer to form the epidermal layer. A fresh HUVEC culture medium was added to the dish. Under submerged culturing conditions, 1.5 mM calcium chloride (CaCl_2_) was added to promote initial cell attachment and proliferation. The medium was replaced with an air–liquid interface (ALI) culture medium containing 50 µg/mL of ascorbic acid (Sigma-Aldrich, St. Louis, MO, USA) and 10 ng/mL of keratinocyte growth factor (KGF; Lonza, Basel, Switzerland) to induce KC differentiation. All media were changed every other day.

### 4.7. Characterization of 3D Skin Model

The printed in vitro skin products were harvested after culturing and fixed with 4% paraformaldehyde for 20 min. They were then subjected to paraffin embedding. Samples were sectioned, mounted on glass slides, and subjected to H&E staining. For immunohistochemical staining, the sections were first deparaffinized. After antigen retrieval in ethylenediaminetetraacetic acid (EDTA) buffer, the sections were then blocked with 3% bovine serum albumin (BSA) for 30 min. The slides were incubated with the primary antibodies anti-cytokeratin 14 (K14, 1:100, Abcam, Cambridge, UK), anti-α-smooth muscle actin (α-SMA, 1:100, Abcam), anti-cytokeratin 10 (K10, 1:100, Abcam), and anti-platelet endothelial cell adhesion molecule-1 (CD31, 1:100, Abcam) overnight at 4 °C in a humidified chamber. After three PBS washes (10 min each), the slides were incubated with secondary antibodies (goat anti-mouse 1:300, goat anti-rabbit 1:400, Servicebio) at room temperature for 1 h and then subjected to further PBS washes. Nuclei were counterstained with DAPI for 10 min, and the slides were washed three times with PBS before being observed under a fluorescence microscope. The fluorescence intensity levels of K10, CD31, K14, and α-SMA in the immunohistochemical sections were quantitatively analyzed using ImageJ-Fiji software.

### 4.8. qRT-PCR

qRT-PCR was utilized to investigate the effects of nano-ZnO on the dermal layer of the 3D skin model. A total of 1 mg of nano-ZnO was added to 10 mL of PBS and vortexed homogenously to form a 0.01% (*w*/*v*) nano-ZnO mixture. Then, 100 μL of the mixture was quickly applied to the epidermis of each skin model for 12 h. Subsequently, the dermis of the in vitro skin products was collected and digested with collagenase (Engineering for Life, Suzhou, China), and cells were collected via centrifugation.

Fluorescence-activated cell sorting (FACS) was used to sort HUVECs and HSFs. Aliquots of a 200–500 µL cell suspension were incubated with antibodies specific for HUVEC and HSF surface markers (1:200) on ice in the dark. Then, the cells were washed and resuspended in fusion flow cytometer buffer. The target HUVECs and HSFs were sorted based on specific fluorescence signals through the FACS (BD Aria™ fusion, BD, Franklin Lakes, NJ, USA). The sorted cells were collected in centrifuge tubes, and total RNA was extracted using TRIzol and reverse-transcribed into cDNA using a PrimeScript™ kit (TaKaRa Bio Inc., Otsu-shi, Japan). qRT-PCR was performed using TB Green^®^ Premix Ex Taq™ (TaKaRa Bio Inc., Otsu-shi, Japan) on an ABI-ViiA7 system. Key genes involved in apoptosis, inflammation, and oxidative stress were detected. Gene expression levels were calculated using the 2^−ΔΔCt^ method and normalized to β-actin. Primer sequences are listed below:

Caspase3, F 5′-CTCGGTCTGGTACAGATGTCGATG-3′ and

R 5′-GGTTAACCCGGGTAAGAATGTGCA-3′;

Bcl2, F 5′-AGATGTCCAGCCAGCTGCAC-3′ and

R 5′-TGTTGACTTCACTTGTGGCC-3′;

BAX, F 5′-ACCAAGAAGCTGAGCGAGTGT-3′ and

R 5′-ACAAACATGGTCACGGTCTGC-3′;

IL-6, F 5′-GGACAACTCAGGGATGCAAT-3′ and

R 5′-GCAGAAGAGAGCCAACCAAC-3′;

TNF-α, F 5′-CCTCTCTCTAATCAGCCCTCTG-3′ and

R 5′-GAGGACCTGGGAGTAGATGAG-3′;

Nrf2, F 5′-ATGGATTTGATTGACATACTTT-3′ and

R 5′-ACTGAGCCTGATTAGTAGCAAT-3′;

β-actin, F 5′-TCATGAAGTGTGACGTGGACAT-3′ and

R 5′-CTCAGGAGGAGCAATGATCTTG-3′.

### 4.9. Statistical Analyses

Data from triplicate experiments (n = 3) are expressed as mean values ± standard deviation (SD). Group significance was determined using Tukey’s test using Origin Pro 2021. Results were considered significant at * *p* < 0.05, ** *p* < 0.01, and *** *p* < 0.001; *p* > 0.05 was not considered significant.

## Figures and Tables

**Table 1:** List of RHC-MA samples.

RHC-I-MA:RHC-III-MA	Composite
7:3	RHC-MA-a
9:1	RHC-MA-b

## Data Availability

The original contributions presented in the study are included in the article. Further inquiries can be directed to the corresponding author.
